# Cardioprotective effect of *Sanguisorba minor* against isoprenaline-induced myocardial infarction in rats

**DOI:** 10.3389/fphar.2023.1305816

**Published:** 2023-12-21

**Authors:** Azar Hosseini, Atieh Ghorbani, Mohaddeseh Sadat Alavi, Nima Forouhi, Arezoo Rajabian, Samaneh Boroumand-Noughabi, Amirhossein Sahebkar, Ali H. Eid

**Affiliations:** ^1^ Department of Pharmacology, Faculty of Medicine, Mashhad University of Medical Sciences, Mashhad, Iran; ^2^ Pharmacological Research Center of Medicinal Plants, Mashhad University of Medical Sciences, Mashhad, Iran; ^3^ Department of Physiology, Faculty of Medicine, Mashhad University of Medical Sciences, Mashhad, Iran; ^4^ Department of Internal Medicine, Faculty of Medicine, Mashhad University of Medical Science, Mashhad, Iran; ^5^ Department of Pathology, Faculty of Medicine, Mashhad University of Medical Sciences, Mashhad, Iran; ^6^ Biotechnology Research Center, Pharmaceutical Technology Institute, Mashhad University of Medical Sciences, Mashhad, Iran; ^7^ Applied Biomedical Research Center, Mashhad University of Medical Sciences, Mashhad, Iran; ^8^ Department of Basic Medical Sciences, College of Medicine, QU Health, Qatar University, Doha, Qatar

**Keywords:** myocardial infarction, isoprenaline, oxidative stress, cardiotoxicity, herbal medicine, superoxide dismutase

## Abstract

**Introduction:** Oxidative stress is a major instigator of various cardiovascular diseases, including myocardial infarction (MI). Despite available drugs, there is still an increased need to look for alternative therapies or identify new bioactive compounds. *Sanguisorba minor* (*S. minor*) is a native herb characterized by its potent antioxidant activity. This study was designed to evaluate the effect of *S. minor* against isoprenaline-induced MI.

**Methods:** Rats were treated with the hydro-ethanolic extract of the aerial parts of *S. minor* at doses of 100 or 300 mg/kg orally for 9 days. Isoprenaline was injected subcutaneously at the dose of 85 mg/kg on days 8 and 9. Then, the activities of various cardiac injury markers including cardiac troponin (cTnT), lactate dehydrogenase (LDH), creatinine kinase muscle brain (CK-MB), creatinine phosphokinase (CPK), and antioxidant enzymes in serum were determined. Malondialdehyde (MDA) and thiol content were measured in cardiac tissue, and histopathological analysis was conducted.

**Results:** Our results show that isoprenaline increased the serum levels of cTnT, LDH, CK-MB, and CPK (*p <* 0.001) and elevated MDA levels (*p <* 0.001) in cardiac tissue. Isoprenaline also reduced superoxide dismutase (SOD), catalase, and thiol content (*p <* 0.001). Importantly, the extract abolished isoprenaline-induced MI by elevating SOD and catalase (*p <* 0.001), reducing levels of MDA, and diminishing levels of cTnT, LDH, CK-MB, and CPK cardiac markers (*p <* 0.001). Histopathological studies of the cardiac tissue showed isoprenaline-induced injury that was significantly attenuated by the extract.

**Conclusion:** Our results suggest that *S. minor* could abrogate isoprenaline-induced cardiac toxicity due to its ability to mitigate oxidative stress.

## 1 Introduction

Cardiovascular disease (CVD) continues to be a leading cause of morbidity and mortality despite advances in modern medicine. Ischemic heart disease and myocardial infarction (MI) are more common than other CVDs. MI, in particular, is a major contributor to CVD-associated mortality. That is not surprising as MI causes irreversible structural and functional damage to the cardiac tissue (Surekha et al., 2007; Ansari et al., 2019).

There is an increased appreciation of medicinal plants and their phytochemicals both in pharmacological studies and drug development programs (Al Dhaheri et al., 2014; El Hasasna et al., 2015; El Hasasna et al., 2016; Athamneh et al., 2017; Alsamri et al., 2019; Badran et al., 2019; Alsamri et al., 2021a; Mesmar et al., 2021; AlKahlout et al., 2022; Alsamri et al., 2022). Approximately, a third of all drugs approved by the Food and Drug Administration (FDA) over the past 20 years have been obtained from or inspired by natural/herbal compounds/derivatives (Izzo et al., 2020). In traditional medicine, there are several natural products such as extracts, herbomineral, polyherbal formulations, and crude herbals, which can be used for CVD (Upaganlawar et al., 2011; Anwar et al., 2018; Hosseini et al., 2022). For example, the cardioprotective activities of some phytochemicals such as silymarin, quercetin, curcumin, berberine, naringenin, baicalin, resveratrol, and myricitrin have been well documented in various studies (Rao and Viswanath, 2007; Giannouli et al., 2018; Hesari et al., 2021). These beneficial effects are due to the presence of a broad spectrum of antioxidative, antiangiogenic, anti-ischemic, anti-thrombotic, and anti-inflammatory activities (Hesari et al., 2021). Among the attractive reasons for utilizing these natural products are their documented fewer side effects, ease of access, and affordability (Upaganlawar et al., 2011; Al Disi et al., 2015; Anwar et al., 2016; Samaha et al., 2019; Salama et al., 2020).

There are various approaches that could be employed to mimic MI in the laboratory. Examples include ligating the coronary artery (Thang et al., 2018) or administration of a high dose of isoprenaline (ISO), a β-adrenergic agonist. The advantage of using ISO is that it is a non-invasive method that has been shown to exert damage to cardiac myocytes *via* various mechanisms including hypoxia, calcium overload, production of free radicals, and energy depletion (Patel et al., 2010).

Different studies have shown that medicinal herbs or their active compounds can attenuate ISO-induced MI by virtue of their antioxidative capacities (Upaganlawar and Balaraman, 2011). One plant that appears to possess several bioactives which are potent antioxidants is *Sanguisorba. minor* (*S. minor*). This plant belongs to the Rosaceae family and is used in traditional medicine for bleeding, eczema, diarrhea, etc. (Zhao et al., 2017). Reports show that several phytochemicals such as phenols, flavonoids, and terpenoids can be isolated from the *Sanguisorba* genus and are known to abate oxidative milieu (Zhao et al., 2017). In particular, the antioxidant activity of *S. minor* is owed primarily to its bountiful polyphenols. Moreover, the pharmacological profile of *S. minor* includes anti-bacterial, neuroprotective, anti-inflammatory, and anti-cancer properties (Zhao et al., 2017; Finimundy et al., 2020; Ćirović et al., 2021). Given the antioxidant activity of various bioactives in *S. minor*, we undertook this study to determine the potential effects of *S. minor* against ISO-induced MI in rats.

## 2 Materials and methods

### 2.1 Reagents

Isoprenaline, trichloroacetic acid (TCA), 2-thiobarbituric acid (TBA), ketamine, xylazine hydrochloride, and 3-(4,5-dimethylthiazol-2-yl) 2,5-diphenyltetrazolium bromide (MTT) were bought from Sigma-Aldrich (St. Louis, MO, United States). 5,5′-Dithiobis-(2-nitrobenzoic) acid (DTNB) and pyrogallol were obtained from Cayman (Michigan, United States).

### 2.2 Preparation of extracts

*S. minor* was obtained from Quchan, Iran (37.1008° N, 58.5054° E). The collected plant was identified by M.R. Joharchi from the Ferdowsi University of Mashhad. The voucher specimen (No. 45489) was recorded and deposited in the Herbarium of the School of Agriculture, Ferdowsi University of Mashhad, Mashhad, Iran. To this end, we used the aerial parts of the *S. minor* which were collected in the spring.

After drying the plant, the aerial parts were ground to fine powder, and 50 g of the powder was soaked in 200 mL of a hydro-ethanolic solution (50%, v/v) for 48 h at 40°C. Afterward, the extract was dried using a rotary vacuum evaporator at 37 °C (Norouzi et al., 2019). The yield of extract was 20% (w/w).

### 2.3 Animals

Adult male Wistar rats weighing 200–250 g were obtained from the Animal Research Center of the Faculty of Medicine, Mashhad University of Medical Sciences, Mashhad, Iran. Rats were kept in a temperature-controlled environment (22 ± 4°C) with a 12 h day/night cycle. Animals had free access to food and tap water *ad libitum*. All experiments were performed in accordance with the National Institutes of Health (NIH) Guide for the Care and Use of Laboratory Animals and were approved by the Animal Ethics Committee of Mashhad University of Medical Sciences, Mashhad, Iran (IR.MUMS.MEDICAL.REC.1399.426).

### 2.4 Stimulation of MI

MI was induced by administration of ISO (subcutaneously; SC) at the dose of 85 mg/kg daily for 2 days (on days 8 and 9) as previously published (El-Gohary and Allam, 2017).

### 2.5 Experimental protocol

**FIGURE 1:**
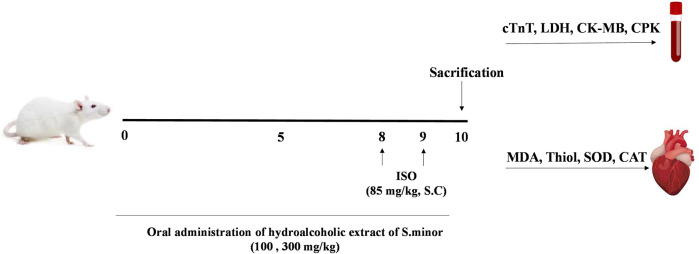
Diagrammatic sketch showing the experimental protocol. ISO: isoprenaline, cTnT: cardiac troponin, LDH: lactate dehydrogenase, CK-MB: creatinine kinase muscle brain, CPK: creatinine phosphokinase, MDA: malondialdehyde, and SOD: superoxide dismutase.

Rats were divided into five groups, each comprising six animals. Saline was gavaged to the control group for 9 days. The ISO group received normal saline for 9 days; ISO was injected SC (0.3 mL) on days 8 and 9. The experimental/therapeutic groups received hydroalcoholic extract orally at the doses of 100 and 300 mg/kg for 9 days; ISO was injected SC (0.3 mL) on days 8 and 9 as performed with the control group. To assess the potential toxicity of the extract, one group of animals received a high dose of the extract (300 mg/kg) for 9 days. For collecting blood samples from the heart, rats were anesthetized with ketamine/xylazine on the last day, and then, the heart tissue was immediately separated and frozen in liquid nitrogen. Tissue and serum samples were stored at −80 °C for further biochemical analyses and histopathological studies (Figure 1).

#### 2.5.1 Measurement of malondialdehyde

Lipid peroxidation was evaluated by the determination of malondialdehyde (MDA) in cardiac tissue as previously published (Boroushaki et al., 2019). In brief, trichloroacetic acid (10%) and thiobarbituric acid (0.67%) were added to the sample and boiled for 40 min. HCl and n-butanol were added to cooled samples. After centrifugation, the absorbance of the supernatant was read at 535 nm. Furthermore, the concentration of MDA was determined as equal to absorbance/(1.56*105 cm^−1^M^−1^).

#### 2.5.2 Determination of thiol contents

The thiol content was measured according to the work of Hosseini et al. (2017). Then, 50 μL of tissue homogenate was mixed with Tris-EDTA buffer (pH = 8.6), and absorbance was measured at 412 nm (A1). 5,5′-Dithiobis-(2-nitrobenzoic) acid (DTNB) was added to the solution, and then, the absorbance after 15 min was determined (A2). DTNB was used as blank (B). The equation used to quantitate the amount of thiol content is total thiol concentration (mM) = ((A2-A1-B)*0.7)/0.05*14.

#### 2.5.3 Determination of catalase activity

Catalase (CAT) activity was measured according to the Aebi protocol (Aebi, 1984). This method is based on the constant rate (k) of hydrogen peroxide destruction detection by measuring its reduction in absorbance at 240 nm/min. The catalase activity was measured as K (constant)/L.

#### 2.5.4 Evaluation of superoxide dismutase activity

The activity of superoxide dismutase (SOD) was determined as previously published (Madesh and Balasubramanian, 1998). In brief, this is a colorimetric assay involving superoxide production by pyrogallol auto-oxidation. Suppression of superoxide-dependent reduction of the MTT by SOD was calculated at 570 nm. A unit of SOD activity is defined as the amount of enzyme that induces 50% inhibition in the MTT reduction rate (Madesh and Balasubramanian, 1998).

#### 2.5.5 Measurement of enzyme markers

The amount of lactate dehydrogenase (LDH), creatinine kinase (CPK), cardiac troponin (cTn-T), creatinine kinase-MB (CK-MB), ALT, and AST was measured in serum according to standard kits as previously published (Lewandrowski et al., 2002).

#### 2.5.6 Measurement of lipid profile

The number of lipids such as total cholesterol (TC), triglyceride (TG), very low-density lipoprotein (VLDL), low-density lipoprotein (LDL), and high-density lipoprotein (HDL) was measured in serum as per the manufacturer’s protocol (Pars Azmun Co., Karaj, Iran).

#### 2.5.7 Histopathological investigation

Formalin (10%) was used to fix isolated cardiac for histopathological studies. The tissues were paraffinized and cut into 3–4 mm slices. Hematoxylin and eosin (H&E) staining was performed, and sections were visualized by light microscopy.

### 2.6 Statistical analysis

All data are expressed as mean ± SEM. One-way ANOVA followed by the Tukey–Kramer test was performed, and a *p* < 0.05 was considered significant.

## 3 Results

### 3.1 Effect of *S. minor* on lipid peroxidation and thiol content

**FIGURE 2:**
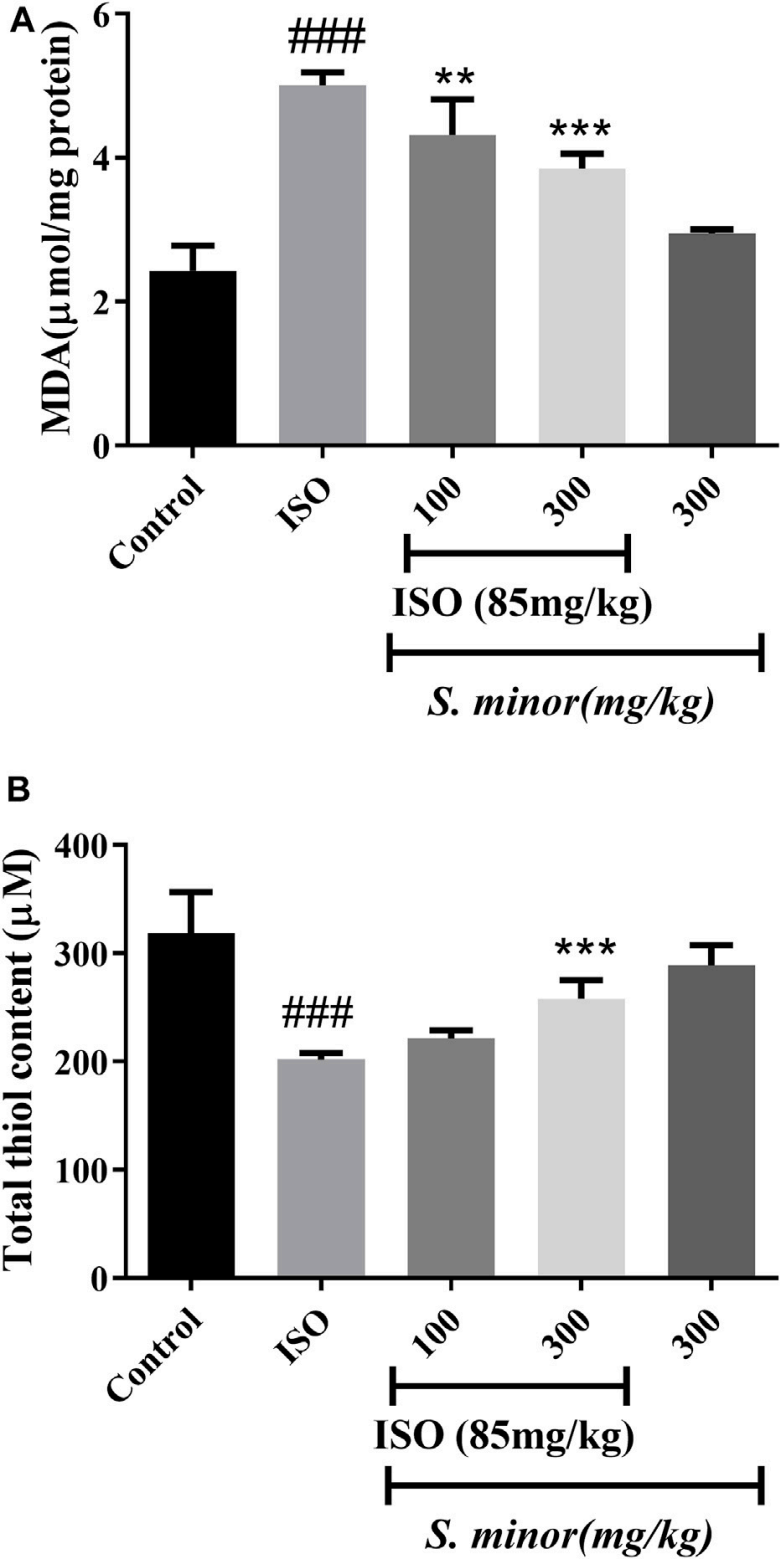
Effect of *S. minor* on the malondialdehyde level and thiol content in heart tissue after isoprenaline administration. The level of MDA **(A)** and thiol **(B)** content was evaluated in heart tissue. Data were expressed as mean ± SEM. ###*p <* 0.001 in comparison with the control group. **p < 0.01 and ***p < 0.001 in comparison with isoprenaline (85 mg/kg).

MDA is considered a lipid peroxidation index. As expected, treatment with ISO increased the level of MDA following MI (###*p* < 0.001). *S. minor* extract significantly reduced the level of MDA at the doses of 100 and 300 mg/kg (**p < 0.01, ***p < 0.001) (Figure 2). On the other hand, ISO decreased thiol content (###*p* < 0.001) in cardiac tissue, while *S. minor* (300 mg/kg) increased the total thiol content (***p < 0.001) (Figure 1). Importantly, the level of MDA and thiol in the extract-only group (i.e., the group without ISO) was similar to that in the control group (Figure 2). Taken together, these findings argue for a cardioprotective effect of *S. minor* against ISO-induced lipid peroxidation or thiol suppression.

### 3.2 Effect of *S. minor* on antioxidant enzymes

**FIGURE 3:**
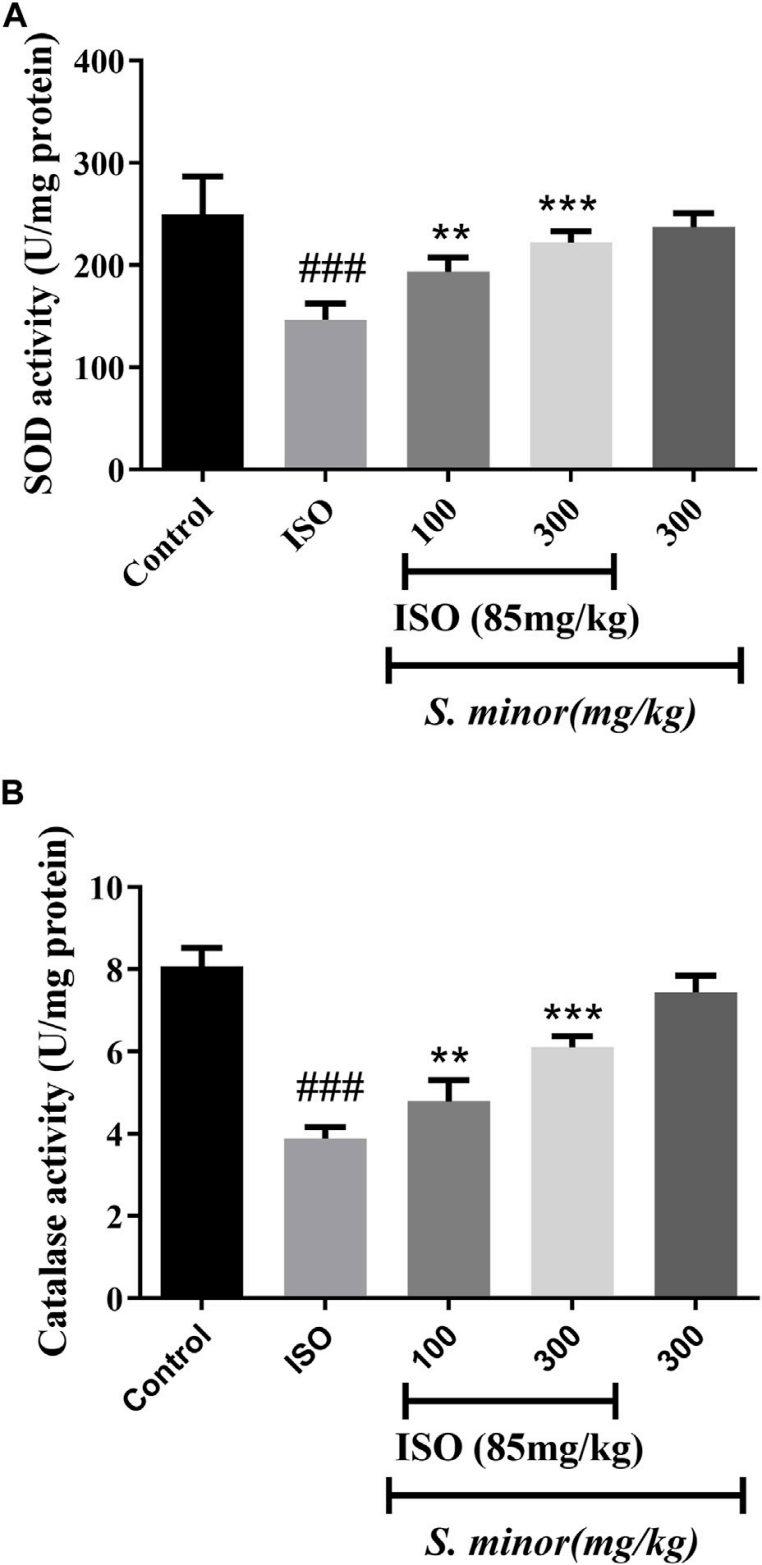
Effect of *S. minor* on the activity of superoxide dismutase and catalase after isoproterenol administration. The activity of SOD **(A)** and CAT **(B)** was evaluated in tissue. Data were expressed as mean ± SEM. ###*p <* 0.001 in comparison with the control group. **p < 0.01 and ***p < 0.001 in comparison with isoprenaline (85 mg/kg).

As shown in Figure 3, the level of the antioxidant enzymes, SOD and CAT, was attenuated following ISO administration (###*p* < 0.001). Importantly, oral pretreatment with *S. minor* at the doses of 100 and 300 mg/kg (**p < 0.01, ***p < 0.001). This result shows that the *S. minor* extract abrogates ISO-induced suppression of antioxidant defense mechanisms.

### 3.3 Effect of *S. minor* on cardiac injury markers

**FIGURE 4:**
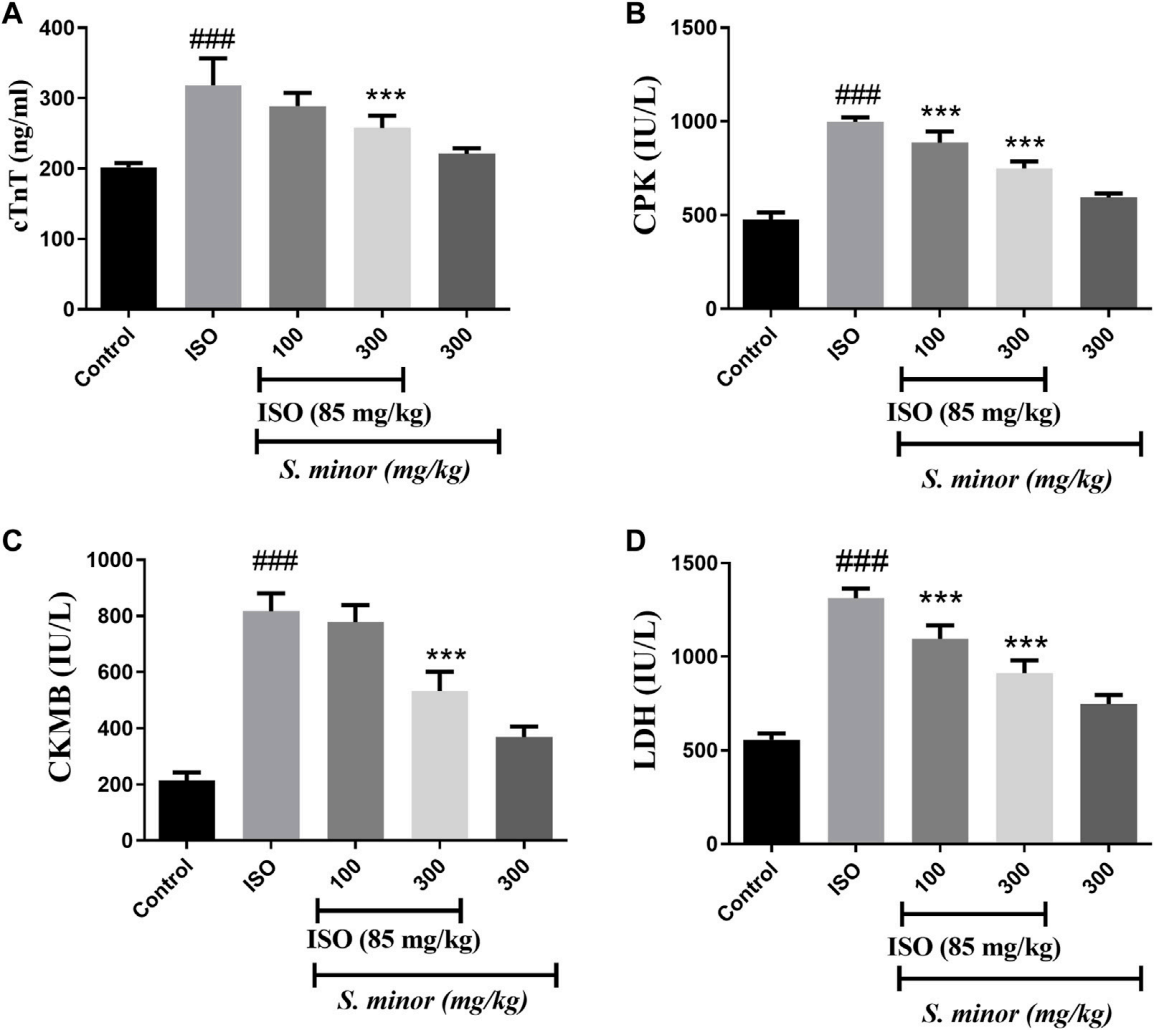
Effect of *S. minor* on the levels of cTn-T, CK-MB, LDH, and CPK after isoproterenol administration. The levels of cTn-T **(A)**, CK-MB **(C)**, CPK **(B),** and LDH **(D)** were evaluated in serum. Data were expressed as mean ± SEM. ###*p <* 0.001 in comparison with the control group. ***p < 0.001 in comparison with isoprenaline (85 mg/kg).

Compared with the control group, animals treated with ISO exhibited significant elevation in serum levels of cTn-T, CPK, CKMB, and LDH (Figure 4) (###*p* < 0.001). Importantly, both doses (100 and 300 mg/kg) of the extract significantly reduced the level of CPK (***p < 0.001) and LDH (***p < 0.001). The levels of CKMB and cTn-T were decreased significantly by the extract at the dose of 300 mg/kg (***p < 0.001). Importantly, the extract alone did not change the level of cardiac injury markers. Thus, these results argue for an ameliorative effect of the *S. minor* extract against cardiac injury.

### 3.4 Effect of *S. minor* on liver enzymes

**FIGURE 5:**
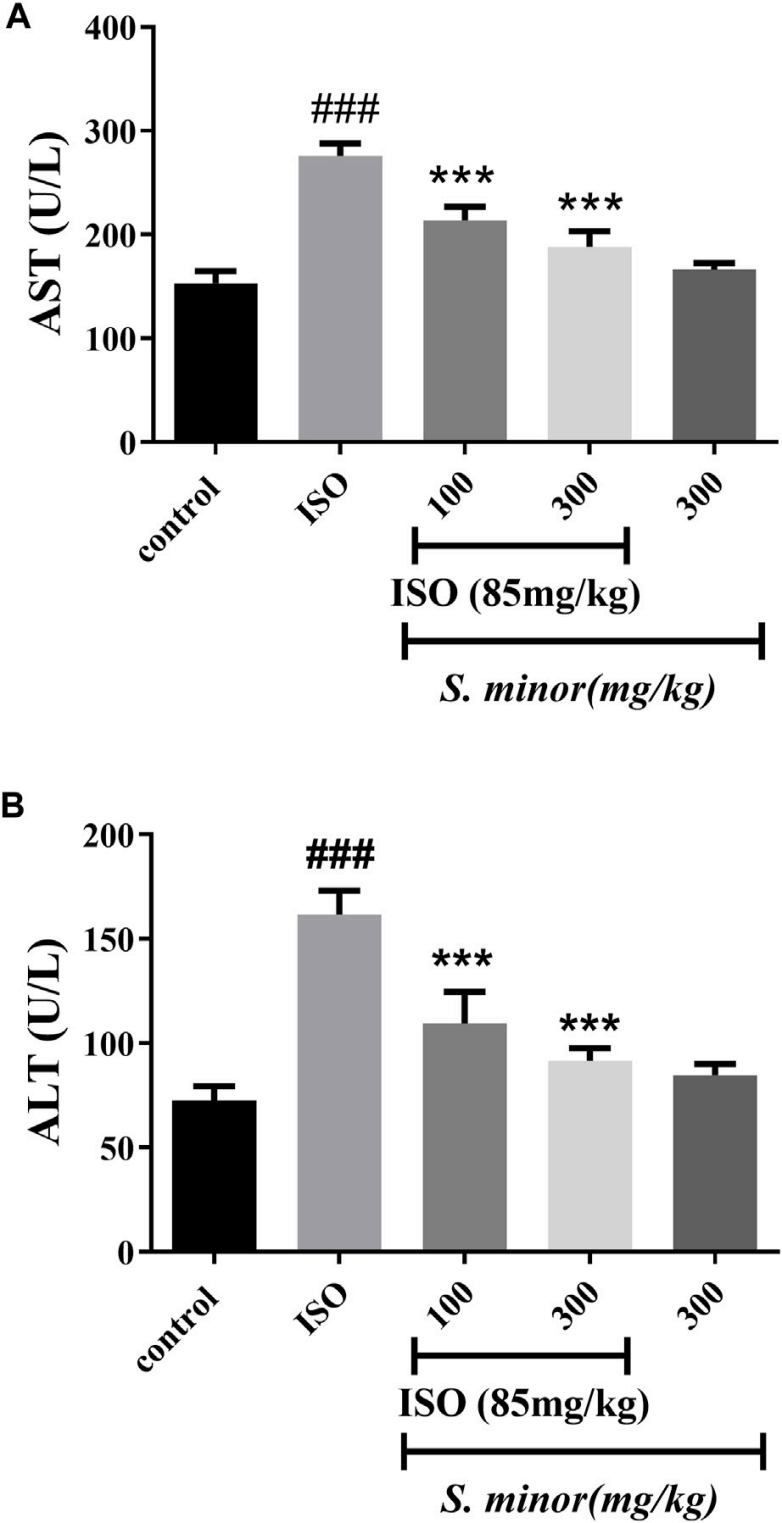
Effect of *S. minor* on the levels of AST **(A)** and ALT **(B)** after isoproterenol administration. The levels of these enzymes were evaluated in serum. Data were expressed as mean ± SEM. ###*p <* 0.001 in comparison with the control group. ***p < 0.001 in comparison with isoprenaline (85 mg/kg).

The serum levels of AST and ALT were measured in serum as shown in Figure 5. ISO caused a dramatic increase in both enzymes in comparison with the control group (###*p* < 0.001). Importantly, 100 mg/kg and 300 mg/kg (***p < 0.001) of *S. minor* attenuated the ISO-induced increase of AST and ALT (Figure 5). It is worth mentioning that the high dose of *S. minor* did not elicit any effects on the levels of both enzymes in the animals treated with the extract alone (i.e., absent of ISO) (Figure 5).

### 3.5 Effect of *S. minor* on the lipid profile

Our results (Table 1) show that ISO increased (###*p* < 0.001) the level of serum lipids following MI induction and decreased the amount of HDL in serum. Oral pretreatment with 300 mg/kg (***p < 0.001) of the extract reduced the amount of TG, TC, LDL, and VLDL, and increased HDL in comparison with the ISO group. A similar effect was noted for 100 mg/kg (*p < 0.05) except that TC was not affected by this dose. Importantly, treatment of *S. minor* alone (i.e., without ISO) did not modify the lipid profile (Table 1). Thus, *S. minor* exerts a favorable effect on the dysregulated lipid profile induced by ISO.

**TABLE 1:** Modulation of the lipid profile by the *S. minor* extract.

Biochemical parameter (mg/dL)	Control	ISO	Extract (100 mg/kg)+ISO	Extract (300 mg/kg)+ISO	Extract alone (300 mg/kg)
TG	72 ± 6.4	218 ± 4.3^###^	199 ± 8*	174 ± 9***	75 ± 7
TC	98 ± 5.4	221 ± 7^###^	210 ± 5	155 ± 4***	100 ± 6.5
LDL	48 ± 2.3	137.5 ± 3^###^	122 ± 5.5*	81 ± 5.2***	51 ± 2.8
VLDL	10.8 ± 0.7	33 ± 0.6^###^	28 ± 0.7*	21 ± 0.4***	11.5 ± 0.78
HDL	27 ± 0.8	19 ± 1.5^###^	21 ± 1.7*	24 ± 0.9***	26 ± 0.88

The levels of TG, TC, VLDL-C, LDL-C, and HDL-C were measured in serum. Data were expressed as mean ± SEM. ###*p <* 0.001 in comparison with the control group. **p < 0.001 and ***p < 0.001 in comparison with isoprenaline (85 mg/kg).

### 3.6 Histopathological effects of *S. minor*

**FIGURE 6:**
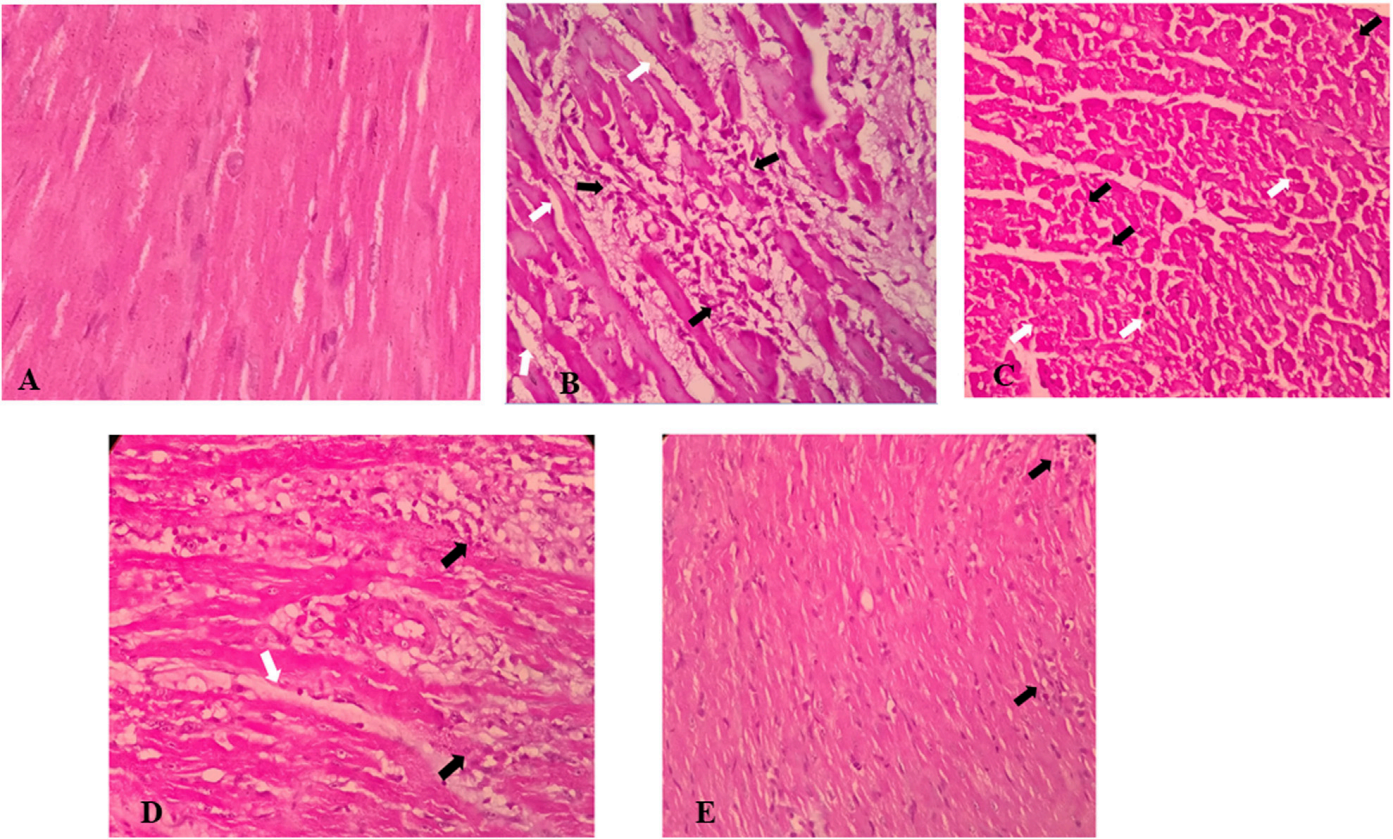
Effects of *S. minor* on histological changes of heart tissue in rats. **(A)** Control group (×400, H&E), **(B,C)** isoprenaline group shows edema (white arrows) and intense inflammatory infiltration (black arrows) **(B)** (×400, H&E) and cellular damage including cytoplasmic hyper eosinophilia (white arrows) and nuclear shrinkage (black arrows) **(C)** (×400, H&E), and **(D)** isoprenaline + 100 mg/kg extract shows edema (white Arrow) and severe inflammatory infiltrate (black Arrow), similar to the isoprenaline group (×400, H&E), while isoprenaline + 300 mg/kg extract [**(E)** ×400, H&E] shows very milder inflammatory infiltration (black arrows).

The microscopic evaluation of the ISO-treated group revealed marked cellular damage including cytoplasmic hypereosinophilia, nuclear shrinkage, edema, congestion, small foci of hemorrhage, and foci of inflammatory cell infiltration composed of macrophages, lymphocytes, and neutrophils (Figure 6). Oral pretreatment with the extract at the dose of 300 mg/kg showed less-pronounced changes including mild edema and congestion, but not hemorrhage, and scarce foci of mild inflammatory reaction (Figure 6). Importantly, treatment with the extract alone at a dose of 100 mg/kg showed some edema and inflammatory infiltrates, while the higher dose (300 mg/kg) exhibited only very mild inflammatory infiltration.

## 4 Discussion

In this report, we employed ISO as an agent to induce a phenotype similar to MI. Our findings showed that ISO increased the levels of TC, TG, LDL-C, and VLDL-C and reduced the amount of HDL-C. This result is in agreement with previous reports showing a dyslipidemic effect of an ISO insult (Manjula et al., 1992). The elevation of lipids in serum following ISO administration in rats might be due to increased mobilization of lipids from adipose tissue or via increased cyclic adenosine monophosphate (Strålfors et al., 1984; Paritha and Shyamala, 1997). Interestingly, recent studies have reported that plants from the Rosacea family can play an ameliorative role in the modulation of the lipid profile (Klevanova and Trzhetsynskiy, 2015; Jung et al., 2016).

In this study, we showed that pretreatment with the extract of *S. minor*, a member of the Rosacea family, significantly reduced serum levels of TC, TG, LDL-C, and VLDL-C, while, at the same time, increasing levels of HDL-C. This favorable modulate of lipidemia by *S. minor* supports its utilization for such an effect. To the best of our knowledge, this is the first study to report the hypolipidemic effects of *S. minor*. Interestingly, however, several studies have previously reported the hypolipidemic effects of quercetin and ellagic acid, two bioactives of *S. minor*. Similarly, studies of other Sanguisorba species such as *Sanguisorba officinalis* also alluded to a hypolipidemic effect. For instance, Mai et al., showed that ellagic acid reduced the lipid profile following ISO administration via HMG-CoA reductase inhibition (Elhemely et al., 2014). Likewise, quercetin decreased the lipid profile following doxorubicin (Aziz, 2021a), and the *S. officinalis* extract modulated the lipid profile in obese mice (Zheng et al., 2023). Based on these studies, it is reasonable to assume that the hypolipidemic effect of *S. minor* is due to its bountiful content of many bioactives, including quercetin and ellagic acid. Although these protective effects of *S. minor* are in accordance with the reported anti-hyperlipidemia effect of this plant family, further research is still needed to better determine and elucidate the underlying mechanisms of *S. minor*.

CK-MB and LDH are two diagnostic markers that are commonly used to determine cardiac injury. They are typically present in the cellular compartment and play a significant role in proper gradients for essential ions across cellular membranes within the myocytes (Preus et al., 1988). Moreover, it is well established that alterations in the properties of these enzymes impart a significant effect on the function of the heart. Contextually, during myocardial damage, CK-MB and LDH leak out into the circulation due to the disintegration of cardiomyocytes (Mari Kannan and Darlin Quine, 2011). It is the stabilization of the sarcolemma that protects cardiomyocytes against injury and, hence, does not allow for dramatic cell damage and release of the aforementioned enzymes. Moreover, as expected, ISO caused a dramatic increase in the levels of several enzymes, namely, CK-MB, CPK, LDH, AST, and ALT. Previous studies have revealed cardiomyocyte injury, resulting from glucose or oxygen deprivation, robustly modified membrane permeability and promoted leakage of these enzymes (Todd et al., 1980). In addition, the cardiac cytosolic calcium level is a key factor involved in maintaining normal activity levels of cardiac enzymes. ISO-induced myocardial necrosis has been reported to enhance adenylate cyclase activity, resulting in increased formation of cAMP and elevation of the cardiac cytosolic calcium level (Suchal et al., 2016).

The levels of LDH, CK-MB, and CK increase after MI and decrease to a normal range within 24 h. Total CK and CK-MB could be related to infarct size and can be used as predictors of prognosis. Elevation of these markers is intimately associated with cell membrane rupture; therefore, stabilization of the membrane by natural products or phytochemicals can have a role in the attenuation of these markers (Aydin et al., 2019). In this study, *S. minor* decreased these markers following ISO-induced MI. We previously reported the characterization of the *S. minor* extract by liquid chromatography–mass spectrometry, which confirmed the presence of quercetin, myricetin, kaempferol, catechin, ellagic acid, and gallic acid derivatives. Here, we show that *S. minor* can be effective in ameliorating the induced infarction, which could be due to the presence of different bioactive ingredients such as quercetin (Panda and Kar, 2015), kaempferol (Vishwakarma et al., 2018a), myricetin (Tiwari et al., 2009), catechin (Devika and Prince, 2008), and ellagic acid (Warpe et al., 2015). These and other flavonoids are well known to exert various beneficial effects including cardiovasculoprotective ones (Fardoun et al., 2019; Maaliki et al., 2019; Fardoun et al., 2020; Alsamri et al., 2021b; Slika et al., 2022). In particular, the cardioprotective effects of quercetin have been widely documented in both *in vitro* and *in vivo* experimental models of cardiac injury. Quercetin exerts cardioprotection in ischemia–reperfusion injury, autoimmune myocarditis, ISO- and doxorubicin-induced cardiotoxicities, and diabetic cardiomyopathy (Arumugam et al., 2012; Chen et al., 2013; Wang et al., 2013; Cote et al., 2015; Kumar et al., 2017). Numerous mechanisms such as suppression of oxidative stress, inhibition of inflammation and apoptosis, and affecting intracellular protein kinase cascades are involved in the cardioprotective potential of quercetin (Li et al., 2016; Ferenczyova et al., 2020). Kaempferol, a flavonoid that is bountiful in *S*. *minor*, could protect against cardiotoxicity precipitated by angiotensin II (Du et al., 2019), cisplatin (Qi et al., 2020), 5-fluorouracil (Safarpour et al., 2022), ISO (Vishwakarma et al., 2018b), or doxorubicin (Xiao et al., 2012). This protection occurs by virtue of the antiapoptotic, antioxidative, anti-inflammatory, calcium regulatory, and antifibrotic mechanisms of kaempferol (Kamisah et al., 2023). Likewise, ellagic acid, another important component of *S*. *minor*, showed cardioprotection against ISO-induced MI by the inhibition of oxidative stress and improvement of cardiac enzymes (Kannan and Quine, 2011).

CTn-T is a contractile protein that controls the calcium-mediated interaction of actin and myosin, which results in contraction and relaxation of striated muscle (Mari Kannan and Darlin Quine, 2011). This protein is not typically expressed in high levels under physiological conditions; however, its levels markedly increased following cardiac necrosis or infarction (Weil et al., 2017). The level of cTn-T after MI has been correlated with the severity of infarction in experimental animal models and contemplated for early detection of cardiotoxic potential in rodents. ISO-caused oxidative damage to the myocardium changes the cell membrane permeability and leads to cTn-T cardiac enzyme leakage to the circulation (Zhang et al., 2008). Here, ISO, and rather expectedly, increased the level of cTn-T, indicative of cellular injury, which was confirmed by Bertinchant et al. (2000). Pretreatment with the extract greatly diminished the increased cTn-T levels and, thus, helped prevent further excessive damage by preserving the structural integrity of the cells.

Oxidative stress plays a critical role in the onset and pathogenesis of many diseases, including CVD (Al Dhaheri et al., 2014; Shouk et al., 2014; Thuan et al., 2018; Alsamri et al., 2019; Mesmar et al., 2021; Shaito et al., 2022; Slika et al., 2022; Aramouni et al., 2023). Our study investigated the level of MDA as an oxidative stress index increased in rats that received ISO. The extract dramatically abrogated the ISO-induced MDA, strongly suggestive of the potent antioxidant activity of *S. minor*. This attenuation by the extract might be due to the elevation of the antioxidant activity of enzymes such as SOD and CAT (Cristina et al., 2021; Ćirović et al., 2021). Superoxide is an antioxidant enzyme that catalyzes the dismutation reaction of superoxide radicals (McCord and Fridovich, 1969). Contextually, the activity of SOD was reduced in ISO-treated rats, while pretreatment with extract restored the activity of SOD. This is in accordance with the report of Shiny et al. (2005) and Shen et al. (2019). Likewise, the activity of catalase decreased in ISO-treated rats in comparison with other groups. The activity of catalase increased following administration of the extract, indicating the antioxidant effects and free radical scavenging potential of the *S. minor* extract (Ćirović et al., 2021). It is well known that MI leads to the accumulation of superoxide radicals in the damaged site, imparting further necrotic effects on the myocardium (Saravanan and Prakash, 2004). Taken together, this shows that the extract decreases the depletion of antioxidant enzymes and, hence, exerts a protective effect against ISO-induced oxidative milieu.

The microscopic evaluation of the ISO-treated group revealed marked cellular damage, edema, congestion, small foci of hemorrhage, and foci of inflammatory cell infiltration composed of macrophages, lymphocytes, and neutrophils. Oral pretreatment of extract at high dose showed less-pronounced changes including mild edema and congestion, but not hemorrhage and scars foci of mild inflammatory reaction. In accordance with our findings, others have shown that ISO induces degenerative changes in heart tissue (Zaafan et al., 2013; Yaseen et al., 2017) and that quercetin improves histopathologic alterations induced by ISO (Zaafan et al., 2013) or doxorubicin (Aziz, 2021b).

The biochemical findings were confirmed by histopathological studies. ISO stimulated degenerative myocardium, inflammation, and hemorrhage which led to more release of cardiac markers, while pretreatment with *S. minor* indicated regenerative effects and attenuated pathological changes. As such, the favorable modulation of the biochemical parameters discussed herewith may, at least partly, explain these beneficial effects of *S. minor* observed in the histopathological findings. Although the present findings provide promising evidence as to the cardioprotective effects of *S. minor* against MI, there are limitations deserving acknowledgment. Assessment of cardiac function, *e.g.,* through echocardiography, was not conducted in this study. In addition, assessment of cardiac stress, inflammation, fibrosis, and endothelial function indices could have added further insights as to the mechanisms underlying the cardioprotective effects of *S. minor* during ISO-induced MI. In a rat ISO model of cardiotoxicity, overstimulation of β-receptors evokes a decrease in the cardiac output and stroke volume and impairs systolic and diastolic functions in echocardiography (Filipský et al., 2012). As such, future studies addressing these limitations may provide better insights into the holistic benefits of *S. minor* in cardiovascular disease, particularly ISO-induced cardiac damage (Filipský et al., 2012).

Finally, it is particularly important here to mention that the content of bioactive compounds varies according to several factors including plant organ, phenological stage, growing regions, climatic patterns, and temperature. For instance, the aerial parts of *S. minor* (leaves and stems) have a high flavonoid content, with quercetin and ellagic acid being predominant, while they are less abundant in roots (Tocai Moţoc et al., 2023). The level of phenolic compounds during spring–summer seasons is higher than in other seasons, which may be related to the production of new current-year leaves (Karkanis et al., 2019). Therefore, careful attention to the content would become important for pharmacotherapeutic applications.

## 5 Conclusion

**FIGURE 7:**
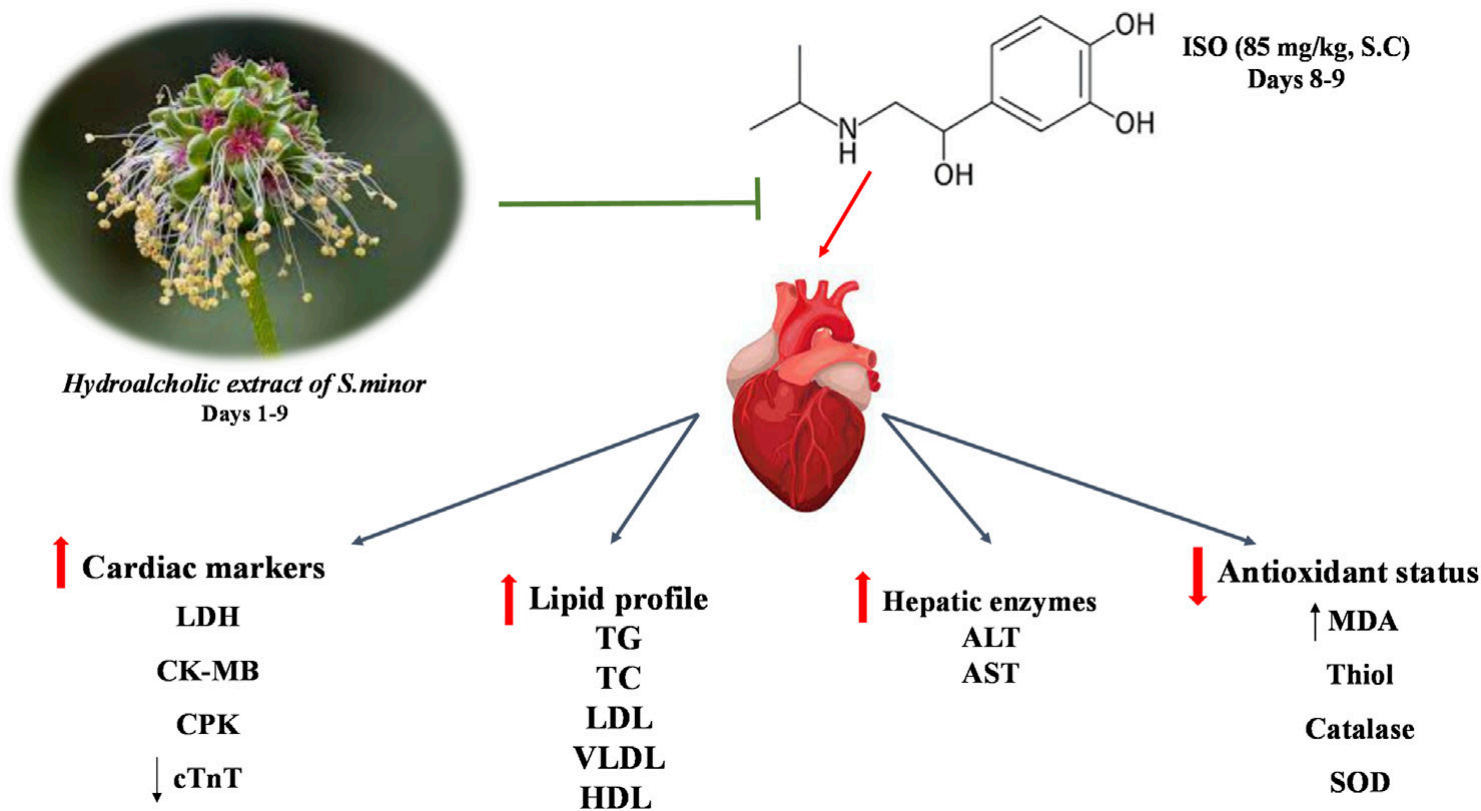
A summary of the key findings.

It is evident from the current study that oral pretreatment with *S. minor* modulated oxidative stress and reduced ISO-induced cardiac injury (Figure 7). The cardioprotective effects of *S. minor* may be linked to the bountiful presence of antioxidant bioactives, such as quercetin, myricetin, kaempferol, catechin, ellagic acid, and gallic acid, in this plant. This supports the traditional use of the plant and adds one more piece of evidence for its incorporation in complementary and alternative approaches in the war against CVD. Given the seasonal variability in the content of the extract and the aforementioned factors contributing to such contents, caution must be taken as the generalizability of findings might be affected by these factors. This necessitates further research on the identification and purification of active ingredients of the plant. Moreover, further studies are certainly needed to absorb this plant or its bioactives into clinical settings.

## Data Availability

TThe raw data supporting the conclusion of this article will be provided by the authors upon a reasonable request.
