# Corrigendum: Cardioprotective effect of *Sanguisorba minor* against isoprenaline-induced myocardial infarction in rats

**DOI:** 10.3389/fphar.2024.1374595

**Published:** 2024-02-21

**Authors:** Azar Hosseini, Atieh Ghorbani, Mohaddeseh Sadat Alavi, Nima Forouhi, Arezoo Rajabian, Samaneh Boroumand-Noughabi, Amirhossein Sahebkar, Ali H. Eid

**Affiliations:** ^1^ Department of Pharmacology, Faculty of Medicine, Mashhad University of Medical Sciences, Mashhad, Iran; ^2^ Pharmacological Research Center of Medicinal Plants, Mashhad University of Medical Sciences, Mashhad, Iran; ^3^ Department of Physiology, Faculty of Medicine, Mashhad University of Medical Sciences, Mashhad, Iran; ^4^ Department of Internal Medicine, Faculty of Medicine, Mashhad University of Medical Science, Mashhad, Iran; ^5^ Department of Pathology, Faculty of Medicine, Mashhad University of Medical Sciences, Mashhad, Iran; ^6^ Biotechnology Research Center, Pharmaceutical Technology Institute, Mashhad University of Medical Sciences, Mashhad, Iran; ^7^ Applied Biomedical Research Center, Mashhad University of Medical Sciences, Mashhad, Iran; ^8^ Department of Basic Medical Sciences, College of Medicine, QU Health, Qatar University, Doha, Qatar

**Keywords:** myocardial infarction, isoprenaline, oxidative stress, cardiotoxicity, herbal medicine, superoxide dismutase

In the published article, there was an error in Figure 4A as published. During the last stage of revision, labels in Figure 4A were inadvertently altered, and the group labels in the figure were mistakenly substituted. The corrected Figure 4A appears below:

**FIGURE 4:**
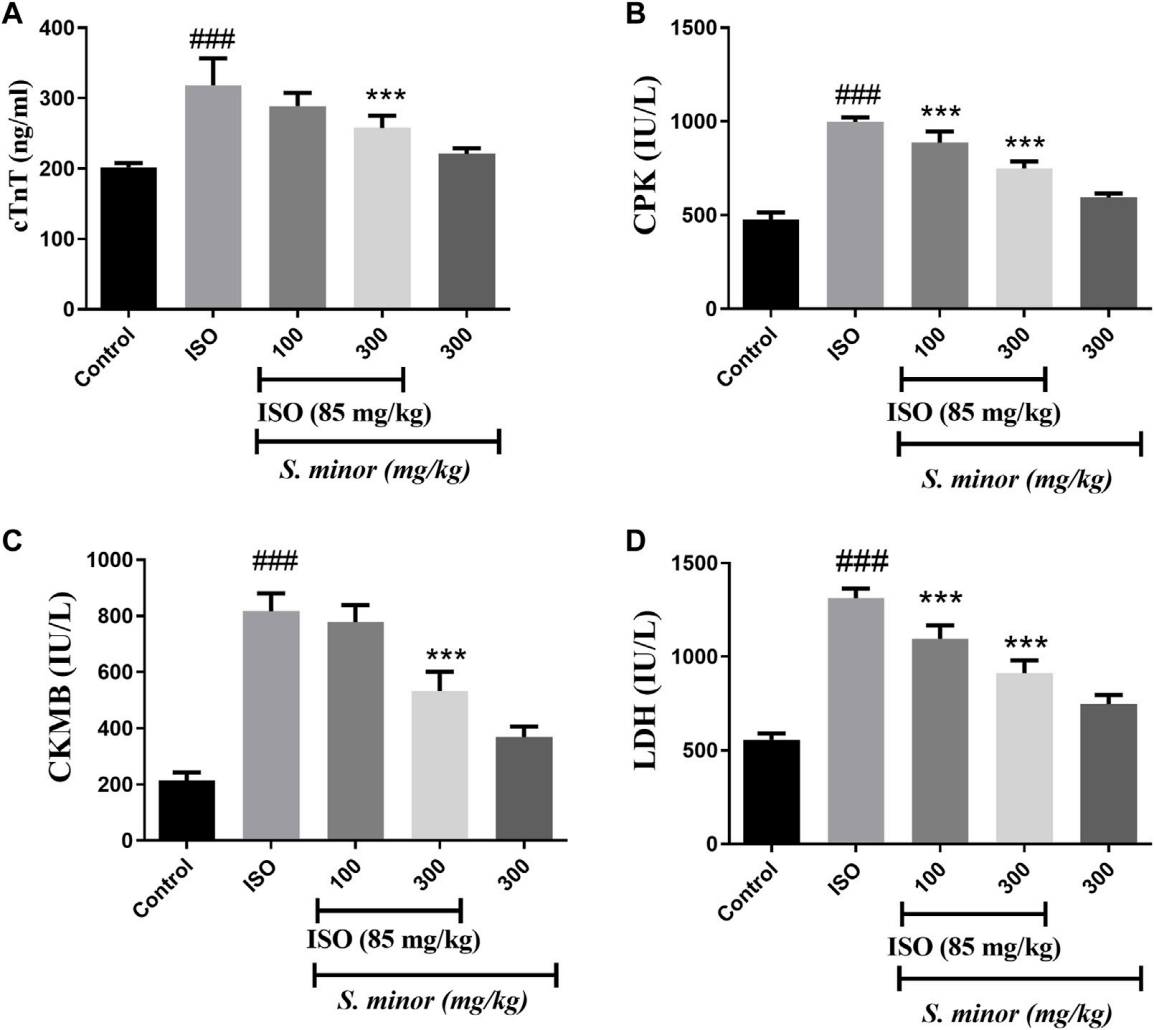
Effect of *S. minor* on the levels of cTn-T, CK-MB, LDH, and CPK after isoproterenol administration. The levels of cTn-T **(A)**, CK-MB **(C)**, CPK **(B)**, and LDH **(D)** were evaluated in serum. Data were expressed as mean ± SEM. ###*p* < 0.001 in comparison with the control group. ***p < 0.001 in comparison with isoprenaline (85 mg/kg).

The authors apologize for this error and state that this does not change the scientific conclusions of the article in any way. The original article has been updated.

